# Ferroptosis in Arthritis: Driver of the Disease or Therapeutic Option?

**DOI:** 10.3390/ijms25158212

**Published:** 2024-07-27

**Authors:** Shania Bieri, Burkhard Möller, Jennifer Amsler

**Affiliations:** 1 Faculty of Medicine, University of Bern, 3012 Bern, Switzerland; 2 Department of Rheumatology and Immunology, Bern University Hospital, University of Bern, 3010 Bern, Switzerland; burkhard.moeller@insel.ch; 3 Department for BioMedical Research DBMR, University of Bern, 3008 Bern, Switzerland

**Keywords:** ferroptosis, ROS, lipid peroxidation, iron, rheumatoid arthritis, osteoarthritis, RA-FLS, chondrocytes

## Abstract

Ferroptosis is a form of iron-dependent regulated cell death caused by the accumulation of lipid peroxides. In this review, we summarize research on the impact of ferroptosis on disease models and isolated cells in various types of arthritis. While most studies have focused on rheumatoid arthritis (RA) and osteoarthritis (OA), there is limited research on spondylarthritis and crystal arthropathies. The effects of inducing or inhibiting ferroptosis on the disease strongly depend on the studied cell type. In the search for new therapeutic targets, inhibiting ferroptosis in chondrocytes might have promising effects for any type of arthritis. On the other hand, ferroptosis induction may also lead to a desired decrease of synovial fibroblasts in RA. Thus, ferroptosis research must consider the cell-type-specific effects on arthritis. Further investigation is needed to clarify these complexities.

## 1. Introduction

Ferroptosis is an iron-dependent form of regulated cell death characterized by lipid hydroperoxide accumulation. The term was coined in 2012 by Stockwell et al. to describe a form of cell death induced by an inhibition of cysteine manifesting an abnormal mitochondrial structure [1,2]. Although extensively studied in cancer cells as a potential therapeutic target [3], its role in autoimmune and autoinflammatory diseases involving arthritis remains less clear despite the fact that iron accumulation in inflamed tissue leads to cellular toxicity. In line with this finding, iron overload has also been found to play a role in the arthritic joint [4]. Further, since the joint remains a hypoxic environment despite the fact that hypoxia-inducible factors induce angiogenesis to some extent, oxidative stress may play an important role in the synovial tissue [5]. Thus, the question arose, whether iron excess and oxidative stress might lead to ferroptosis in the inflamed joint. Alternatively, iron chelation is experimentally used as a mimic of hypoxia response and could serve as an alternative explanation for several findings in arthritis [6]. In recent years, the relationship between inflammation and ferroptosis has been explored in several ways for a better understanding of diverse pathomechanisms in inflammatory diseases, since ferroptosis is accompanied by the release of pro-inflammatory molecules, such as interleukin (IL)-1β and IL-18 [7]. Thus, research on ferroptosis in diseases involving arthritis has taken its path in order to find new therapeutic targets for these in part still not adequately treatable diseases.

This review aims to elucidate the role of ferroptosis in arthritis and its therapeutic implications. For the search conducted in this review, we used the title and abstract key terms ““ferroptosis” and “arthritis” or “rheumatoid arthritis” or “osteoarthritis” or “spondylarthritis” or “cristal arthropathies” or “gout”” in PubMed.

### 1.1. Regulating Ferroptosis

**Figure 1:**
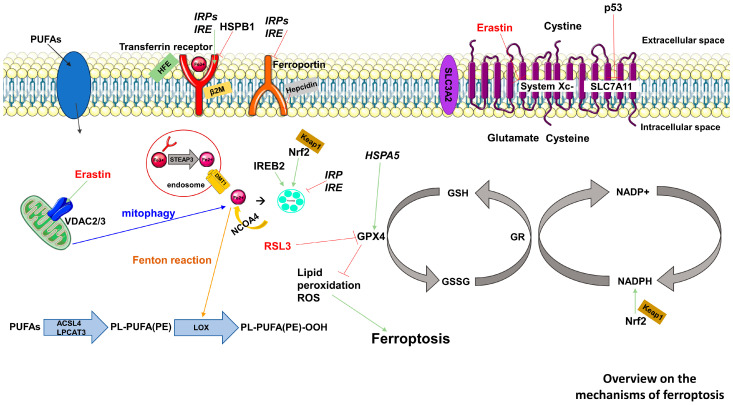
General overview of the mechanisms of ferroptosis, irrespective of the cell type. System Xc- functions as an amino acid antiporter, widely distributed within phospholipid bilayers. Comprising two subunits, solute carrier family 7 member 11 (SLC7A11) and solute carrier family 3 member 2 (SLC3A2), it forms a crucial part of the cellular antioxidant system. The exchange of cystine and glutamate occurs through System Xc- at a balanced ratio of 1:1, both entering and exiting the cell. Cystine, acquired through cellular uptake, undergoes reduction within cells and participates in glutathione (GSH) synthesis. GSH plays a role as an electron donor in reducing reactive oxygen species (ROS) and reactive nitrogen under the influence of GPXs, thereby forming oxidized GSSG out of two GSH molecules. Hampering the function of system Xc- influences the synthesis of GSH by impeding cystine absorption, leading to diminished GPX activity, reduced cellular antioxidant capacity, lipid ROS accumulation, and oxidative damage, culminating in ferroptosis. Further abbreviations are explained in the abbreviation table at the end of the review.

Ferroptosis is distinct from other forms of regulated cell death like apoptosis, pyroptosis, necroptosis, and autophagy. It is primarily driven by excessive intracellular iron and dysregulated lipid repair systems, leading to lipid peroxidation and morphological changes in mitochondria [8]. During ferroptosis, the activity of glutathione peroxidase 4 (GPX4) is reduced, which appears to play a crucial role. Lower GPX4 expression increases susceptibility to ferroptosis, while higher expression is inhibiting [9,10] (Figure 1).

RAS-selective lethal 3 (RSL3) targets and suppresses GPX4 activity, leading to lipid ROS accumulation and ferroptosis induction [11]. Erastin, initially identified for its selective toxicity in tumor cells, has gained attention as a ferroptosis inducer. Erastin triggers ferroptosis through various mechanisms involving system XC-, voltage-dependent anion channels, and p53 [12].

### 1.2. Iron Metabolism

Iron accumulation is a key trigger of cytotoxic processes disrupting redox balance and leading to cell death [13]. In the serum, transferrin serves as the primary iron transport protein [14]. Iron-bound transferrin (Fe^3+^) may bind to transferrin receptor 1 (TfR1), which is followed by uptake of complexed Fe^3+^ into cells [3]. Within endosomes, STEAP3 converts Fe^3+^ to ferrous iron (Fe^2+^), which is released into the labile iron pool (LIP) via divalent metal transporter 1 (DMT1, also known as SLC11A2). Most cytoplasmic Fe^2+^ binds to ferritin light (FTL) and ferritin heavy chain (FTH), forming ferritin, mediated by chaperones like Poly-(rC)-binding protein 1 (PCBP1) and PCBP2 [3]. Iron export is facilitated by FPN, the sole mammalian iron efflux pump [14]. Ferroptosis-sensitive cells with Ras mutations show elevated TfR1 and reduced ferritin expression, suggesting that increased iron uptake and decreased iron storage contribute to iron overload during ferroptosis. Iron chelators like deferoxamine reduce iron overload and inhibit ferroptosis induced by molecules like erastin, while supplementing iron increases erastin-induced cell death. Knockdown of iron-responsive element-binding protein 2 (IREB2) increases expression of iron metabolism genes (e.g., FTH1 and FTL), inhibiting ferroptosis. Thus, as uptake and utilization systems of iron critically regulate ferroptosis, the availability of this metal is itself an essential component of the cellular response [14].

### 1.3. Lipid Peroxidation Pathways

Two critical metabolites for ferroptosis arise from lipid peroxidation, lipids and reactive oxygen species (ROS), which are primarily generated through the Fenton reaction [15]. Free polyunsaturated fatty acids (PUFAs) may act as substrates in the synthetic lipid signaling pathway but must be esterified into membrane phospholipids and oxidized to transmit the ferroptotic signal. Arachidonic acid (AA)-containing phosphatidylethanolamine (PE) is a key phospholipid with the capacity to induce ferroptosis. Acyl-CoA synthetase long-chain family member 4 (ACSL4) and lysophosphatidylcholine acyltransferase 3 (LPCAT3) participate in PE biosynthesis and remodeling, activating PUFAs and influencing PUFA’s transmembrane characteristics [16]. In the non-enzymatic pathway, hydroxyl radicals (HO) strip lipids of hydrogen atoms (H∙) to form lipid radicals, which create lipid peroxides [17]. In the enzymatic pathway, iron-dependent lipoxygenases (LOXs) convert PUFAs into peroxides and derivatives like malonaldehyde (MDA) and 4-hydroxynonenal (4-HNE), both leading to ferroptosis [16]. Lipid peroxidation can impair membrane-bound enzymes, altering plasma membrane fluidity and permeability [18].

## 2. Ferroptosis in Inflammatory Arthropathies

Joint inflammation (arthritis) is a hallmark of most rheumatic and musculoskeletal diseases (RMDs), which substantially affects the patient’s body function and quality of life [19]. There are more than 100 different, more specifically described clinical entities, all of which may go along with arthritis, but they importantly differ in their etiology, non-arthritic symptoms, severity, chronicity, and long-term outcome. Some types of arthritis have a known etiology like bacteria, crystals, or viruses, but the vast majority of arthritis is currently of unknown nature. Treatment of arthritis has been focused on certain immunological cell types and pro-inflammatory cytokines; however, there is an unmet need for other treatment avenues, since more than 50% of patients with immune-mediated arthritides do not respond satisfactorily to current treatment options [20]. At present, researchers are increasingly recognizing potential connections between inflammatory arthritis and ferroptosis. This exploration holds promise in elucidating unknown components in the pathogenesis of inflammatory arthropathies, while unveiling prospective therapeutic targets for the future.

In this review, we will focus on RA, spondylarthritis (SpA), psoriatic arthritis (PsA), and crystal arthropathies, followed by a chapter about osteoarthritis (OA).

Table 1 summarizes current in vivo data from ferroptosis research in animal models with arthritis and from isolated and in vitro stimulated cell types from rats and mice. In addition, Table 2 summarizes ex vivo data observed in stimulated and non-stimulated human tissue from selected arthritis conditions.

### 2.1. Ferroptosis in Resident Joint Cells: Fibroblast-Like Synoviocytes (FLSs) and Synovial Membrane

In a healthy joint, the synovium is a delicate structure that covers the inner surface of articular joints, regulating synovial fluid for smooth movement. The synovium consists of two layers: the lining layer and the sublining layer. The lining layer, in direct contact with synovial fluid, comprises spindle-shaped fibroblasts and macrophages, providing a barrier and releasing lubricants like hyaluronic acid. The sublining layer contains less densely packed fibroblasts, macrophages, and blood vessels within a loose tissue matrix [83]. According to a concept of arthritis starting inside the synovium, initial events would happen in the resident cells in the synovium. In arthritis, particularly rheumatoid arthritis (RA), the synovial lining layer undergoes significant hyperplasia, with fibroblast-like synoviocytes (FLSs) expanding and adopting an activated phenotype, infiltrating damaged cartilage and bone [84,85,86]. The sublining layer also expands with infiltrating inflammatory cells like macrophages, T cells, and B cells [87]. By producing cytokines, chemokines, and extracellular matrix components associated with disease progression, FLSs play a key role in joint destruction [88]. In established inflammation, activated FLSs release a plethora of cytokines and chemokines, thereby recruiting B cells and T cells to form ectopic germinal centers.

Researchers identified ferritin proteins (FTL, FTH) and iron transport proteins (like TfR and DMT1) in FLSs and macrophages from RA synovial tissue. They found that IL-6 and TNF-α enhance iron uptake by monocytes and FLSs from RA patients in vitro [68,73]. The review will further explore the role of ferroptosis in FLSs within the context of RA.

### 2.2. Ferroptosis in Innate Immune Cells

#### 2.2.1. Polymorphonuclear Neutrophils (PMN)

Neutrophils, essential cells of innate immunity, play a pivotal role in initiating and advancing arthritis. They are the most abundant immune cell in the synovial fluid of most types of arthritis but are only a minor population in synovial tissue. Previously viewed as a homogeneous cell population, we now recognize different neutrophil subsets with varying properties, even including immunosuppressive types [89,90]. In arthritis, neutrophils experience increased cell survival and oxidative stress, thereby continuing to release neutrophil contents and form extracellular traps. These actions together with their interaction with other immune cells sustain inflammation and contribute to joint cartilage and bone degradation [91]. Notably, despite their fundamental role, there is currently no published research on neutrophil ferroptosis in arthritis.

#### 2.2.2. Macrophages and Dendritic Cells (DC)

There is evidence indicating that the accumulation of iron in inflammatory lesions worsens arthritis by triggering ferroptosis in macrophages. A positive correlation exists between elevated levels of iron in synovial fluid and the severity of RA, akin to the correlation observed between lipid hyperoxidation in specific macrophage populations and RA disease severity. Further investigation revealed that anti-inflammatory macrophages (M2) are highly susceptible to iron-induced ferroptosis, whereas pro-inflammatory macrophages (M1) are less affected. The ferroptosis inhibitor liproxstatin has been shown to mitigate the progression of K/BxN serum-transfer-induced arthritis in mice, accompanied by a shift towards M2 macrophages [50].

### 2.3. Ferroptosis in Adaptive Immune Cells

#### 2.3.1. B Lymphocytes (B Cells)

B cells play a crucial role in linking the development of tertiary lymphoid tissue in inflamed synovium to the autoimmune process of RA, supported by the presence of germinal-center-like structures and the impact of B-cell-derived lymphotoxin-α on lymphoid architecture. CD4 T cell activation in the synovium depends on the presence of B cell follicles, with depletion of B cells hindering interferon-γ and IL-1 production, suggesting that other antigen-presenting cells cannot substitute for B cells in maintaining T cell activation [92]. To date, there has been no research on B cell ferroptosis in arthritis.

#### 2.3.2. T Lymphocytes (T Cells)

The synovium in the joints of patients with arthritis harbors various types of immune cells, with monocytes/macrophages and T cells being pivotal components. Monocytes/macrophages have the ability to attract and stimulate the differentiation of T cells into inflammatory phenotypes within the synovium. Similarly, distinct subtypes of T cells can attract monocytes/macrophages, fostering osteoclast differentiation and triggering the production of inflammatory cytokines [93]. In RA synovial tissue, CD4 T cells were found to be lower compared to control synovial tissue, whereas the presence of CD8 T cells was increased [94]. The only study found about T cell ferroptosis in arthritis showed that low doses of the neuroleptic haloperidol can suppress T cell ferroptosis in RA by decreasing the buildup of ferrous ions within lysosomes, resulting in a decreased production of intracellular ROS [95].

### 2.4. Rheumatoid Arthritis (RA)

RA is an immune-mediated inflammatory type of arthritis starting on some genetic background and possibly triggered by environmental factors. Clinically overt RA often occurs many years after the first detection of autoantibodies, indicating that currently unknown factors, other than autoantibodies and genes, are fundamental in this disease [96].

#### 2.4.1. Iron Metabolism in RA

A study revealed disparities in iron metabolism between RA patients and healthy controls. In contrast to the less inflammatory type of arthritis in iron overload disease, disturbances in iron metabolism are not known as the primary event of immune-related arthritis. In contrast, probably secondary iron deficiency is prevalent (64%) among RA patients with elevated disease activity. RA patients exhibited decreased levels of hepcidin, transferrin saturation, and ferritin [97]. It was also found that RA patients had notably elevated serum soluble transferrin receptor (sTfR) levels alongside significantly lower serum iron levels compared to the control group. sTfR demonstrated a significant positive correlation with parameters of inflammatory activity and autoimmune disease [98]. One study examined the serum-derived proteomic alterations in patients classified as non-responders and responders 14 weeks after receiving a combination treatment of methotrexate + leflunomide + infliximab. Results revealed that serum transferrin levels were reduced at baseline in the non-responder group but elevated in the responder group. Further analysis suggests that serum transferrin plays a role in the hypoxia-inducible factor (HIF)-1 pathway and ferroptosis, potentially influencing the therapeutic outcome of this triple therapy [99].

#### 2.4.2. Ferroptosis in RA

**Figure 2:**
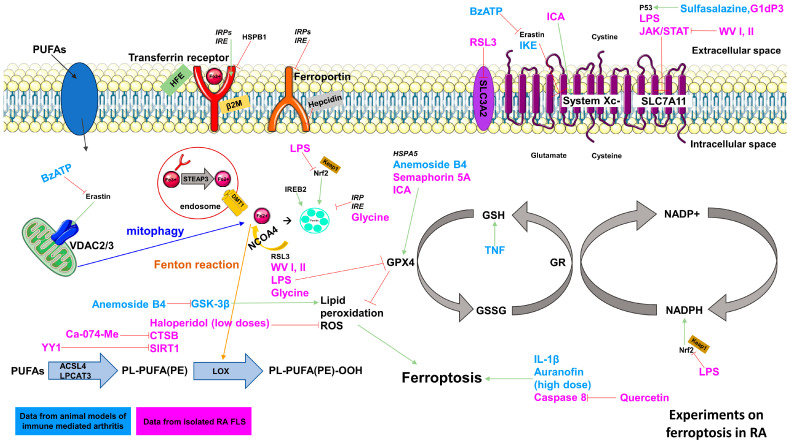
Experiments on ferroptosis in rheumatoid arthritis. Animal experiments are marked in blue. Experiments on isolated human fibroblast-like synoviocytes (FLS) are marked in pink. Abbreviations are explained in the glossary.

The impact of ferroptosis in RA is mainly dependent on the cell type undergoing ferroptosis. Here, we concentrate on the FLS, which are not only resident cells and candidates for the initial events of arthritis, but well-established key players in the RA joint and spreading of RA [100] (Figure 2).

The central genes implicated in ferroptosis within RA synovium potentially include vascular endothelial growth factor A (VEGFA), prostaglandin endoperoxide synthase 2 (PTGS2), and JUN (transcription factor JUN), which are primarily associated with the FoxO signaling pathway [94]. PTGS2, enolase 1 (ENO1), and granulin (GRN) were pinpointed and confirmed as plausible biomarkers associated with the regulation of ferroptosis. It was noticed that knocking down ENO1 resulted in heightened production of lipid ROS, greater buildup of intracellular ferrous ion, and increased cell mortality, as well as higher expression of aconitase 1 (ACO1). Research unveiled ENO1’s elevated expression in RA synovium and suggested that ferroptosis might be governed by the ENO1–ACO1 axis [64]. Another study screening the hub genes in RA found caspase-8 to be a significant biomarker for ferroptosis in RA as it was significantly increased in the ferroptosis phenotype group compared to the control group. Quercetin, a naturally occurring flavonoid, which exhibits strong antioxidant properties and has notable anti-inflammatory effects, can lower the caspase-8 levels, suggesting that it could be a potential treatment target for RA [101].

#### 2.4.3. Ferroptosis Inducers in RA-FLS

Researchers found that imidazole ketone erastin (IKE) decreased FLS populations in CIA mice, leading to reduced concentrations of GPX4 in the remaining synovial tissue [25]. On the other hand, sustained exposure to TNF-α, implicated as a key driver in RA pathogenesis, protected RA-FLSs from ferroptosis by enhancing cystine uptake and glutathione (GSH) synthesis. Conversely, erastin-induced ferroptosis worsened joint inflammation and disrupted gut microbiota and metabolites in CIA mice. Administering an antagonist 2′(3′)-O-(4-Benzoylbenzoyl) adenosine 5-triphosphate (BzATP) alleviated arthritic inflammation and abnormal intestinal microbiota caused by erastin [31]. These results suggest that novel ferroptosis inducers used in combination with established TNF inhibitors could potentiate the therapy for RA.

Experiments with RA-FLSs treated with RSL3 showed decreased expression of solute carrier family 2 member 3 (SLC2A3), FTH1, SLC7A11, and GPX4, thereby inducing ferroptosis along with increased lipid peroxidation and accumulation of ferrous ions [74].

Cathepsin B (CTSB), identified as a promising RA biomarker, was inhibited by CA-074-methyl ester (CA-074Me), reducing RA-FLS proliferation and migration through lipid ROS, as well as ferrous ion accumulation, and decreasing levels of FTH1, SLC7A11, and GPX4, ultimately leading to ferroptosis [66].

Lipopolysaccharide (LPS) stimulation decreased GPX4, SLC7A11, 4F2 cell-surface antigen heavy chain (SLC3A2L), and Nrf2 expression in human synovial cells. Serum analysis of RA patients revealed decreased levels of Sirtuin 1 (SIRT1) but increased levels of Yin Yang 1 (YY1). SIRT1 enhanced cell viability and reduced ROS and iron concentrations in LPS-induced FLS, while YY1 reversed these effects [76,78,102].

Sulfasalazine, used in inflammatory bowel disease and inflammatory arthritis, triggered ferroptosis in RA-FLSs by activating PI3K-AKT-extracellular signal-regulated kinase (ERK)1/2 and p53-SLC7A11 pathways [30,54].

Moreover, glycine was found to enhance ferroptosis in RA-FLSs by decreasing GPX4 levels and reducing FTH1 expression [68].

Additionally, vespa magnifica venom promoted ferroptosis in human rheumatoid FLSs by decreasing GPX4 levels [75].

Furthermore, galectin-1-derived peptide induced ferroptosis in TNF-α-stimulated rheumatoid FLS cells by enhancing lipid peroxides and iron deposition and suppressing SLC7A11 expression [67].

Conversely, small extracellular vesicle (sEV) production from RA-FLSs increased during ferroptosis induction due to local inflammation, led to elevated synovial VEGF expression, and enhanced angiogenesis. The release of sEV during ferroptosis may be linked to compensatory upregulation of the endosomal sorting complex required for transport (ESCRT-III), which aids in repairing cellular damage from ferroptosis stimulation. LPS-treated FLSs showed increased ESCRT-III levels, and knockdown of the ESCRT-III subunit CHMP4A increased ROS levels and decreased GPX4 and SLC7A11 concentrations [70]. This study highlights the importance of a repair system that has to be taken into account when inducing ferroptosis in FLS.

These diverse approaches of ferroptosis induction in RA-FLSs suggest that targeting synovial proliferation through ferroptosis may offer a new therapeutic avenue distinct from conventional immune-based treatments.

#### 2.4.4. Ferroptosis Inhibitors in RA-FLS

Although ferroptosis inhibition in RA-FLSs is not a goal in view of their role in the disease, studies investigating ferroptosis inhibition in RA-FLSs have revealed interesting results, including the following.

Semaphorin 5A activates the PI3K/AKT/mTOR pathway in FLS, increasing GPX4 expression and preventing ferroptosis [82]. Icariin (ICA), from herba epimedii, reverses RSL3-induced ferroptosis in FLS, restoring cell viability and reducing lipid peroxidation and iron accumulation. ICA also protects FLSs from LPS-induced cell death by activating the Xc-/GPX4 pathway [102]. Furthermore, ICA reduces bone loss due to iron buildup and protects mice osteoblasts by reducing iron overload and inhibiting apoptosis [48]. Nuclear receptor coactivator 4 (NCOA4) drives ferroptosis in RA-FLSs by mediating ferritinophagy. Inhibiting NCOA4-mediated ferritinophagy protects RA-FLSs from ferroptosis triggered by LPS-induced inflammation under hypoxic conditions [54].

These ferroptosis inhibitors might not be relevant for RA-FLSs but could possibly be used for other cell types such as chondrocytes in RA or OA.

Inducing ferroptosis in fibroblast-like synoviocytes (FLS) could shrink the synovial pannus, thus leading to the resolution of inflammation. However, the exact role of ferroptosis in RA is not fully established. Further detailed studies are needed to understand how the mechanism of ferroptosis can be utilized in the treatment of RA.

### 2.5. Spondylarthritis (SpA) and Other Arthritic Forms

Spondylarthritis (SpA) is another group of inflammatory diseases that primarily affect the joints in the spine. It has a similar morphology to synovitis but is linked to other genetic predispositions than RA. One type of SpA is psoriatic arthritis (PsA) [103,104].

PsA has more similarities with RA than other types, as it may also lead to bone erosion, but it has new bone formation in common with other types of SpA. Similarly to RA, the pathogenesis of PsA is largely influenced by proinflammatory cytokines, with key players including TNF-α and various interleukins, which have significant effects on joint structure [105].

#### 2.5.1. Ferroptosis in Spondylarthritis

Only one published paper has focused on ankylosing spondylitis (AS) and ferroptosis. It demonstrated that patients with AS can be categorized into two distinct subtypes using ferroptosis-related genes (FRG)-based consensus clustering analysis. These subgroups exhibited clear differences in FRG expression patterns, as well as variations in immune cell compositions and enrichment of differentially expressed genes (DEGs) in pathways associated with mitochondria and ubiquitin [106]. To analyze DEGs between these groups, the research team identified 12 hub genes and constructed a multifactorial regulatory network. Notably, the key nodes within this network were closely linked to redox homeostasis and the musculoskeletal system. The study indicates a potentially significant role of ferroptosis in the pathogenesis and molecular regulation of AS [107]. However, further research in this area is needed to determine whether ferroptosis can be targeted in SpA.

#### 2.5.2. Ferroptosis in Psoriatic Arthritis

Only one study has investigated ferroptosis in PsA, focusing on the association between ferroptosis regulators and key genes linked to PsA. The findings revealed a unique relationship between CDGSH iron sulfur domain 1 (CISD1), a ferroptosis regulator, and C-type lectin domain family 2 member B (CLEC2B), a hub gene, in individuals with PsA [108]. Further exploration is needed to determine if targeting this pathway could have therapeutic implications for PsA.

### 2.6. Ferroptosis in Crystal-Induced Arthritis

Crystal arthropathies in the case of gout and pseudogout are characterized by the accumulation of monosodium urate or calcium pyrophosphate dihydrate (CPPD) crystals in joints and surrounding tissues, leading to inflammation [109].

Research has validated the observed connection between higher levels of serum ferritin, iron, and elevated levels of serum urate. This provides evidence that elevated serum ferritin levels are positively linked to an increased risk of gout and more frequent gout flares. Mendelian randomization has shown evidence indicating a causal link between ferritin and iron in raising urate levels, but not the other way around. From a clinical perspective, the data imply that advising people with gout to avoid iron-rich foods could potentially help reduce the frequency of gout flares [110]. According to a large-scale population study across China, there was a positive correlation between serum ferritin, TfR levels, and serum uric acid levels, as well as the likelihood of hyperuricemia [111]. Iron’s role in gout could be linked to xanthine oxidase (XO), which generates uric acid. Research indicates that iron might enhance the expression and function of XO [112]. Additionally, certain cytokines like TNF-α and IL-6 could stimulate XO activation in bovine renal epithelial cells, leading to ROS production [113]. It has been found that monosodium urate crystals can trigger various types of cell death, including ferroptosis, ultimately resulting in inflammatory cell death [114].

Currently, there is no study examining the impact of ferroptosis on crystal arthropathies, although evidence from epidemiological and mechanistic studies concerning the involvement of iron in gout suggest that ferroptosis could be an interesting pathway to investigate.

## 3. Ferroptosis in Osteoarthritis

**Figure 3:**
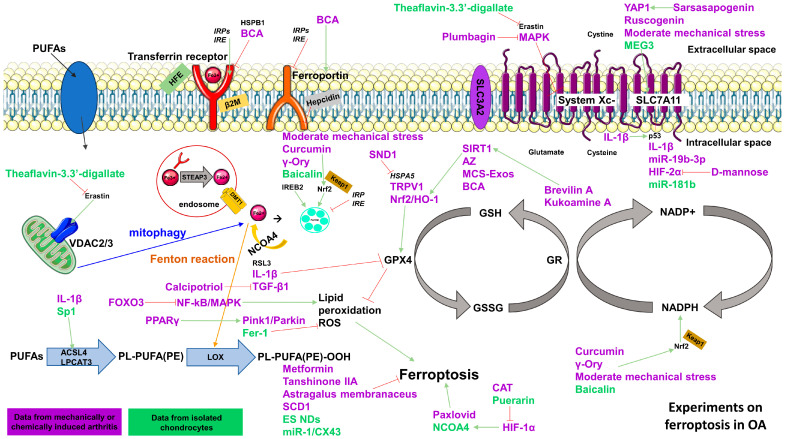
Experiments on ferroptosis in osteoarthritis. Animal experiments for inhibition or induction of ferroptosis in vivo or in vitro are marked in purple. Experiments on human chondrocytes in vitro are marked in green. Abbreviations are explained in the glossary.

OA is a form of arthritis that has been primarily linked to age-related wear and tear. In recent years, OA has been recognized as a multifaceted condition involving cartilage breakdown, bone remodeling, and joint inflammation. Risk factors include advancing age, female gender, obesity, prior joint injuries, anatomical abnormalities, and familial predisposition [115]. The disruption of iron homeostasis is linked to cellular ferroptosis and degenerative diseases [72]. Serum iron levels are higher in OA patients compared to controls, but transferrin expression and total iron binding capacity are diminished [39]. Ferroptosis research in OA primarily targets chondrocytes, a cell type with an unequivocally central role in OA development (Figure 3). Very briefly, cartilage is composed of a dense network of collagen fibers embedded in an aggrecan gel, with a sparse population of cells known as chondrocytes. Chondrocytes play crucial roles in matrix production, repair, and remodeling. They respond to various signals, including growth factors, cytokines, and biomechanical forces to maintain cartilage health. Aging chondrocytes have an important impact on cartilage biology and pathology, affecting tissue function and resilience. Unlike other tissues, cartilage lacks mechanisms for cell replacement, making it vulnerable to irreversible damage from inherited factors and environmental stressors like trauma and obesity [36,116].

Researchers identified ferroptosis-related genes as diagnostic biomarkers and therapeutic targets for synovitis in OA, including EGFR. Inhibition of EGFR induced chondrocyte ferroptosis and matrix degradation, which was reversed by ferrostatin [60,79]. NCOA4 expression is elevated in OA cartilage, aged mice, and mice with post-traumatic OA, driven by c-JUN N terminal kinase (JNK)-JUN signaling. Knocking down NCOA4 in IL-1β-treated chondrocytes reduced ferroptosis markers such as ACSL4 and p53, while increasing GPX4 and cell viability [72].

Another ferroptosis-related marker is sterol carrier protein 2 (SCP2), which is elevated in OA chondrocytes. SCP2 facilitated ferroptosis by transporting lipid peroxidation products to mitochondria, leading to cartilage degradation. Treating OA with a SCP2 inhibitor mitigated cartilage degradation and increased cell viability [29]. Furthermore, long non-coding RNA maternally expressed 3 (lncRNA MEG3) was reduced in OA synovial fluid. Silencing lncRNA MEG3 decreased chondrocyte viability and increased ferroptosis markers. In contrast, increasing MEG3 reduced ferroptosis by modulating the miR-885-5p/SLC7A11 signaling pathway [60]. Additionally, miRNA-1 expression was lower in OA cartilage compared to healthy cartilage, and its upregulation prevented ferroptosis in OA chondrocytes in a mouse model [53]. Moreover, a study showed that stearoyl-CoA desaturase (SCD1) deficiency, a rate-limiting enzyme in the synthesis of unsaturated fatty acids, induced ferroptosis in chondrocytes. SCD1-ko mice developed early OA spontaneously, which further exacerbated in accelerated joint destruction after destabilization of the medial meniscus (DMM), an established model of mechanically induced OA [58]. Conversely, in another set of experiments in the DMM mouse model, cyclin-dependent kinase inhibitor 1A (CDKN1A = p21) expression was increased in OA chondrocytes and in erastin-treated cells. However, p21 knockdown aggravated OA and exacerbated ferroptosis. Thus, p21 appears to exert a crucial anti-ferroptotic function in OA by modulating the stability of GPX4 [42].

Inflammatory and fibrocartilage chondrocytes in osteoarthritic cartilage showed activation of the ferroptosis pathway, along with increased iron-overload-related gene expression [117]. This observation was confirmed by another study observing increased iron concentration, lipid peroxidation, and ferroptotic driver expression in damaged OA cartilage compared to intact areas. Single-cell RNA sequencing identified a distinct ferroptotic chondrocyte cluster, proposing transient receptor potential vanilloid 1 (TRPV1) as a potential anti-ferroptotic target in OA cartilage. Activating TRPV1 protected chondrocytes from ferroptosis and mitigated OA progression in a mouse model [59]. Further, exosomal miR-19b-3p and miR-181b from OA-FLS, found to be increased in the cartilage of OA patients, promoted ferroptosis in mice chondrocytes, highlighting a potential link between synovium, cartilage, and ferroptosis in OA [27,71].

Interestingly, mechanical strain triggered ferroptosis in chondrocytes via piezo-type mechanosensitive ion channel component 1 (piezo1)-mediated calcium influx. Inhibiting piezo1 mitigated mechanical damage and ferroptosis [26]. However, moderate mechanical stress slowed cartilage deterioration in a rat model and suppressed ferroptosis-related genes by triggering the Nrf2 antioxidant system [80]. Thus, the amount of mechanical stress might play a role in the induction of ferroptosis in cartilage.

### 3.1. Ferroptosis Inhibition in IL-1β Stimulated Chondrocytes

In vitro OA modeling often involves IL-1β stimulation of chondrocytes. IL-1β stimulation suppresses GPX4 and SLC7A11 expression while increasing p53, ACSL4, and ROS levels in murine chondrocytes. Treatment with ferrostatin (Fer-1), a ferroptosis inhibitor, reverses these effects by increasing GPX4 and SLC7A11 levels, reducing p53 as well as ACSL4 expression and enhancing cell viability and proliferation [24]. Similar findings were confirmed in another study showing decreased chondrocyte viability with IL-1β treatment compared to IL-1β and Fer-1 treatment [118]. IL-1β treatment also upregulated specificity protein 1 (Sp1), a transcription factor involved in ACSL4 transcription, promoting ferroptosis [65]. Furthermore, activating transcription factor 3 (ATF3) and transferrin receptor (TFRC) were upregulated in IL-1β-stimulated human chondrocytes, while CXCL2, JUN, lysophosphatidylcholine acyltransferase 3 (LPCAT3), and phosphogluconate dehydrogenase (PGD) were downregulated. Elevated ROS levels were also observed in IL-1β-treated chondrocytes compared to controls [63,69].

Similarly, increased expression of staphylococcal nuclease domain-containing 1 (SND1) in IL-1β-stimulated chondrocytes led to GPX4 degradation. Silencing SND1 in these chondrocytes and in a DMM rat model reduced TNF-α levels and iron concentrations and increased GPX4 expression [28].

Likewise, puerarin, from pueraria lobata root, increased chondrocyte viability, decreased inflammatory cytokines, and interacted with ferroptosis mechanisms in IL-1β-treated chondrocytes [81]. Meanwhile, gamma-oryzanol (γ-Ory) from rice bran oil reduced extracellular matrix breakdown and prevented ferroptosis by interacting with Keap1 and Nrf2 binding sites in IL-1β-stimulated rat chondrocytes [47].

These studies highlight IL-1β as a possible trigger for ferroptosis induction.

### 3.2. Ferroptosis Inhibition in Unstimulated Chondrocytes

In human cartilage explants, cellular markers of ferroptosis were positively correlated with the severity of cartilage damage and MMP-13 expression. When chondrocytes from mild OA cartilage were treated with Fer-1, cells showed improved activity and mitochondrial function and lower MMP13 expression, increased concentrations of GPX4 as well as SLC7A11, but diminished ACSL4 and p53 levels, whereas cells from moderate to severe OA could not be rescued in their function by ferroptosis inhibition [44]. This is an interesting finding suggesting ferroptosis might play a role in the beginning of the disease.

Further, epigallocatechin-3-gallate-based nanodrugs (ES NDs) alleviated ferroptosis-induced oxidative stress and reduced inflammation and cartilage breakdown while stimulating cartilage formation. ES NDs decreased iron, lipid peroxidation, and ACSL4 expression but increased FTH1 and GPX4 levels [62].

Moreover, mesenchymal-stem-cell-derived exosomes (MCS-Exos) reduced ferroptosis in murine chondrocytes by increasing GSH and GPX4 levels, suppressing iron accumulation, and stimulating Nrf2/HO-1 expression [51]. Another study showed similar results in rat IL-1β-stimulated chondrocytes [36].

There are several studies exploring the effect of plant extracts on ferroptosis inhibition in OA chondrocytes. In a mouse model, curcumin, an extract derived from turmeric rhizomes, prevented cartilage breakdown induced by erastin. Silencing Nrf2 reversed the beneficial effects of curcumin, suggesting a role in enhancing chondrocyte resistance via inhibition of ferroptosis [40]. Similarly, acetyl zingerone (AZ), a curcumin derivative, increased chondrocyte viability and proliferation in another DMM model, apparently suppressing ferroptosis by promoting GPX4 expression and Nrf2/HO-1 activation [41]. Likewise, theaflavin-3,3′-digallate, extracted from black tea, protected rat and human chondrocytes against erastin-induced ferroptosis via Nrf2/GPX4 pathway activation [32].

Further, kukoamine A, an extract from lycium chinense, reduced articular cartilage loss and MMP expression, preventing chondrocyte death through the SIRT1/GPX4 pathway [49]. MMP expression was also reduced by brevilin A, derived from centipeda minima, which exhibited protective effects against OA by increasing GPX4 through the SIRT1/Nrf2 signaling pathway [37]. Moreover, biochanin A (BCA), an extract from the huangqi plant, reduced intracellular iron levels, inhibited expression of TfR1, and activated Nrf2/system Xc-/GPX4 signaling to neutralize free radicals and inhibit lipid peroxidation in a DMM mouse model [119]. Another plant derivate, ruscogenin, isolated from radix ophiopogon japonicus, protected cartilage by increasing GSH, GPX4, and Nrf2 expression via the Nrf2/SLC7A11/GPX4 pathway [35]. Capsiate (CAT), a metabolite from intestinal microorganisms, inhibited HIF-1α and activated solute carrier family 2 member 1 (SLC2A1) to impede ferroptosis in a DMM model [39]. Another approach targeting hypoxia-inducing factors is D-mannose, which reduced cartilage degradation by targeting HIF-2α-mediated chondrocyte susceptibility to ferroptosis [43].

Further, sarsasapogenin, a steroidal sapogenin, reduced cartilage degradation and increased GPX4 expression in a rat model [46,57]. In a temporomandibular OA rat model, plumbagin, which possesses anti-inflammatory properties, protected cartilage by upregulating GPX4 and controlling MAPK signaling pathways [56]. Additionally, FoxO3 can mitigate the progression of OA by ferroptosis through the nuclear factor (NF)-kB/mitogen-activated protein kinase (MAPK) signaling pathway [45].

Astragalus membranaceus (AM), a Mongolian plant, and tanshinone IIA, a salvia miltiorrhiza derivate, increased GPX4 and SLC7A11 levels to mitigate ferroptosis in murine chondrocytes [34,120].

Concerning drugs, calcipotriol, a synthetical vitamin D analogue, reduced cartilage damage by inhibiting GPX4-mediated ferroptosis and suppressing TGF-β1 and lipid peroxidation of chondrocytes in a DMM mouse model [38]. The antidiabetic drug metformin mitigated OA-associated histopathological changes and abnormal angiogenesis in subchondral bone in a DMM model [52], while pioglitazone, a peroxisome-proliferator-activated receptor γ (PPARγ) agonist, increased GPX4 via PTEN-induced kinase 1 (Pink1)/Parkin-dependent mitophagy to protect RSL3-treated rat chondrocytes from ferroptosis [55]. Conversely, paxlovid, a drug that inhibits the synthesis of virus-related proteins and replication of viral RNA, induced ferroptosis in chondrocytes and accelerated their senescence and degeneration in a mouse model of OA [121].

In summary, inhibiting ferroptosis in OA chondrocytes appears to be beneficial for the outcome of several models of OA. However, the pharmacological compounds appear to be very heterogeneous, and in particular, the potentially active compounds in the phytopharmacological intervention are not well described. Thus, further investigation is needed to determine if any of these approaches may be effective and safe in humans as well.

## 4. Interpretation of the Data

To summarize the results of our review, we found accumulating evidence that ferroptosis might be a key event in the initiation of cartilage pathologies. In ex vivo studies on human cartilage explants, in metabolically or mechanically induced models of OA in rodents, we found consistent evidence that ferroptosis might be a relevant component in the breakdown of cartilage. Thus, it appears to be desirable to pharmacologically inhibit ferroptosis in early OA. However, OA-related interventions were heterogeneous, the sample size of the studies is usually small, and data are not yet clear enough to propagate a certain intervention. This is particularly true for most of the smaller studies with plant-derived compounds.

However, while inhibition of ferroptosis appears to be desirable in resident cartilage cells to prevent OA, the ferroptosis inducer erastin was able to improve inflammatory arthritis severity in CIA mice [31,40,50]. It appears that in this model of arthritis, inhibition of ferroptosis in FLS, another type of resident joint cells (like chondrocytes), might have detrimental effects.

Thus, it is currently not fully clear whether ferroptosis might be a physiological and beneficial process for tissue integrity, or a critical step in arthritis pathogenesis worth being treated. From the current literature, the impact of ferroptosis appears to strongly rely on mechanical or immunological stimuli and cell type. The current literature indicates that inhibiting ferroptosis in anti-inflammatory macrophages, chondrocytes, and T-cells leads to reduced inflammation in the joints of rheumatoid arthritis (RA) and osteoarthritis (OA) and related models. Conversely, inducing ferroptosis in RA fibroblast-like synoviocytes (FLS) appears to have a positive effect, alleviating inflammation.

## 5. Conclusions and Future Perspectives

Although the current state of research suggests an important role for ferroptosis in arthritis, the origins of the literature are currently not globally distributed but restricted mostly to Asian countries. Additionally, confirmation studies are often missing. However, this scarcity of research may be due to the relatively young nature of this topic. We identified a relevant gap in the research on ferroptosis in some prevalent and well-defined human diseases with known pathogenesis, such as crystal-induced arthritis. Moreover, given the major role of FLSs despite only being established in the pathogenesis of RA, it is tempting to speculate that the effect of inhibiting ferroptosis on the severity of synovitis in SpA and PsA might be fundamentally different from RA.

A limitation of our review may be that the search terms were restricted to title or abstract words, possibly leaving out some studies. Writing this review, we realized that ferroptosis induction or inhibition on a whole organism is restricted to rodents, thus leaving us with only cell-type-specific studies for human data. Since the still-preliminary results on the effect of ferroptosis in the development of RA and OA appear to demonstrate opposite consequences in disease-relevant cell types, we assume that ferroptosis does not explain the development of arthritis in general but can account for certain aspects of its pathogenesis.

In summary, the involvement of ferroptosis in different types of arthritis is cell-type-specific and requires careful evaluation for new treatment approaches. Further in vivo studies are needed to understand how ferroptosis can be utilized in the treatment of RA and OA. Given the consistent data on chondrocyte biology and the limited treatment options, especially in OA, targeting ferroptosis in chondrocytes might be a promising strategy to reduce the global burden of this prevalent disease in the aging population.

## Figures and Tables

**Table 1:** Effects of induction and/or inhibition of ferroptosis in animal models with arthritis. In vitro studies concentrate on rodent chondrocytes and synovial fibroblasts. They demonstrate that induction of ferroptosis in synovial fibroblasts by different agents decreases the viability of synovial fibroblast and GPX4 expression. On the other hand, inhibition of ferroptosis in chondrocytes increases cell viability and decreases lipid peroxidation and cartilage degradation. ↑: upregulated; ↓: downregulated; →: results in.

Experiments in Animals	In Vivo	In Vitro
**Inducer of ferroptosis**		
Auranofin	${Hfe}^{-/-}$ mice + auranofin → died within 42 d Auranofin + Fer-1 → cell viability↑, PTGS2↓, thioredoxin reductase↓. Wild type mice + thioredoxin reductase inhibitor → lipid peroxidation↑, PTGS2↑ → Fer-1 could counteract these effects [21]	
Erastin		Articular rat chondrocytes + erastin → cell viability↓, SLC7A11↓, FTH↓, GPX4↓, cytotoxicity↑, TRPM7↑, ACSL4↑, COX2↑ [22]
IL-1β		ATDC5 cells + IL-1β → lipid peroxidation↑, MDA ↑, NCOA4↑, LDH↑, FTH↓ [23]
IL-1β		Mice chondrocytes + IL-1β → GPX4↓, SLC7A11↓, p53↑, ACSL4↑, ROS↑ IL-1β + Fer-1 → GPX4↑, SLC7A11↑, p53↓, ACSL4↓ [24]
Imidazole ketone erastin (IKE)	CIA mice + IKE → RA-FLS↓, GPX4↓ [25]	
Mechanical overload		Chondrocytes from wild type mice + intense mechanical stress by activation of piezo1 → calcium influx↑, ferroptotic damage↑, ROS↑, GSH↓ Mechanical stress + GsMTx4 → calcium influx↓, ferroptotic damage↓, ROS↓, GSH↑ [26]
OA-FLS exosomes	OA model group → miR-19b-3p↑, iron concentrations↑, ACSL4↑, GSH↓, GPX4↓, SLC7A11↓ OA-FLS exosomes → MDA↑, ACSL4↑, iron concentrations↑, GSH↓, GPX4↓, SLC7A11↓ [27]	Chondrocytes + IL-1β + Exo → miR-19b-3p↑, MDA↑, ACSL4↑, ROS↑, cell viability↓, GSH↓, GPX4↓, SLC7A11↓ Chondrocytes + miR(+) + Exo → cell viability↓, GPX4↓, SLC7A11↓, GSH/GSSG ratio↓, MDA↑, ROS↑, ACSL4↑, iron concentrations↑ Chondrocytes + IL-1β + miR(+) → cell viability↓, GPX4↓, SLC7A11↓, GSH↓, MDA↑, ACSL4↑, ROS↑, iron concentrations↑ Chondrocytes + IL-1β + miR(−) → cell viability↑, GPX4↑, SLC7A11↑, GSH↑, MDA↓, ACSL4↓, ROS↓, iron concentrations↓ Chondrocytes + IL-1β + miR(+) + SLC7A11 → cell viability↑, GPX4↑, SLC7A11↑, GSH↑, MDA↓, ACSL4↓, ROS↓, iron concentrations↓ [27]
Staphylococcal nuclease domain containing 1 (SND1)	OA rats + sh-SND1 → GPX4↑, HSPA5↑, TNF-α↓, MDA↓, iron concentrations↓, cartilage tissue damage↓ [28]	Chondrocytes + sh-SND1 → HSPA5↑, GPX4↑, TNF-α↓, ROS↓, MDA↓, iron concentrations↓ Chondrocytes + sh-SND1 + sh-HSPA5 → HSPA5↓, GPX4↓, TNF-α↑, ROS, ↑ MDA↑, iron concentrations [28]
Sterol carrier protein 2 (SCP2)	Hulth + SCP2 inhibitor → cartilage degradation↓, OARSI↓ score, iron concentrations↓, ACSL4↓, SCP2↓, MDA↓ [29]	Chondrocytes + RSL3 or SCP2 inducer or RSL3 + SCP2 inducer → SCP2↑ Chondrocytes + RSL3, RSL3 + SLCP2 → SCP2/VDAC proportion on mitochondria↑ Chondrocytes + RSL3 + SCP2 inducer → MDA↑, MMP-13↑, ROS↑, lipid hydroperoxides levels↑ Chondrocytes + RSL3 + SCP2 inhibitor → MDA↓, MMP-13↓, ROS↓, lipid hydroperoxides levels↓, cell membrane rupture↓, SCP2/VDAC proportion on mitochondria↓ [29]
Sulfasalazine	CIA mice + sulfasalazine → GPX4↓, SLC7A11↓ [30]	
**Inhibitor of ferroptosis**		
2′(3′)-O-(4-Benzoylbenzoyl) adenosine 5-triphosphate (BzATP)	CIA mice + erastin + BzATP → severity of arthritis↓, joint destruction↓, SLC7A11↑ [31]	
Acetyl zingerone (AZ)	OA mice + AZ → cartilage healing↑, GPX4↑, bone deterioration↓ [32]	Rat chondrocytes + IL-1β + AZ → cell viability↑, cell proliferation↑, GPX4↑, COX2↓, MMP-13↓, MDA↓, morphological mitochondria alterations↓ [32]
Anemoside B4	CIA mice + anemoside B4 → GSK-3β activity↓, pain↓ via GSK-3β/Nrf2, ROS↓, NLRP3↓ [33]	
Astragalus membranaceus (AM)	OA mice + AM → MMP-13↓, IL-1β↓, IL-6↓, TNF-α↓, GPX4↑, SLC7A11↑ [34]	
Biochanin A (BCA)	OA mice + biochanin A → iron accumulation↓, cartilage erosion↓, Nrf2↑ [35]	Mice chondrocytes + biochanin A → cell viability↑, HO-1↑, Nrf2↑, iron accumulation↓, ROS↓ [35]
Bone marrow mesenchymal stem cell-derived exosomes (BMSC-Exos)	OA mice + BMSC-exos → OARSI↓, iron concentration↓, MDA↓, METTL3↓, ACSL4↓, GSH↑ [36]	Rat chondrocytes + IL-1β + exo → cell viability↑, GSH↑, iron concentration↓, MDA↓, ROS↓, METTL3↓, m6A↓ [36]
Brevilin A	OA mice + brevilin A → MMP-1↓, MMP-3↓, COX2↓ [37]	Chondrocytes + IL-1β + brevilin A → PGE2↓, MMP-1↓, MMP-3↓, MDA↓, iron concentrations↓, GSH↑, GPX4↑, SIRT1↑, Nrf2↑, HO-1↑ [37]
Calcipotriol	OA mice + calcipotriol → MMP-13↓, TGF-β1↓, GPX4↑ [38]	Chondrocytes + IL-1β + calcipotriol → ROS↓, lipid peroxidation↓, TGF-β1↓, GPX4↑ [38]
Capsiate (CAT)	OA mice + CAT → MDA↓, H_2_O_2_↓ OA mice + HIF-1α agonist or SLC2A1 agonist → MMP-3↑, MMP-13↑, COL2↑ HIF-1α inhibitor or SLC2A1 inhibitor → MMP-3↓, MMP-13↓, COL2↓ [39]	
Curcumin	Mice + erastin + curcumin → breakdown of cartilage↓, cartilage damage↓, MMP-9↓, MMP-13↓, aggrecan↑, collagen II↑, SLC7A11↑, GPX4↑, FTH1↑ Mice + erastin + curcumin + shNrf2 → breakdown of cartilage↑, cartilage damage↑, MMP-9↑, MMP-13↑, aggrecan↓, collagen II↓, SLC7A11↓, GPX4↓, FTH1↓ [40]	Chondrocytes + curcumin → LDH↓, MDA↓, iron concentrations↓, ROS↓, ACSL4↓, GPX4↑, SLC7A11↑, FTH1↑, Nrf2↑ Chondrocytes + erastin + curcumin + si-Nrf2 compared to erastin + curcumin → LDH↑, iron concentration↑, ACSL4↑, TFR1↑, GPX4↓, SLC7A11↓, FTH1↓, Nrf2↓ [41]
Cyclin-dependent kinase inhibitor 1 (p21)	P21↑ in OA model of mice than in sham group [42]	Chondrocytes + IL-1β + erastin → p21↑ Knockdown p21 → proliferation rate chondrocytes↓, GSH↓, MDA↑, ROS↑, iron concentrations↑, lipid peroxidation↑ [42]
D-mannose	OA mice + D-mannose → cartilage degradation↓, MMP-13↓, HIF-2α↓, cartilage degeneration↓, MDA↓, collagen II↑, GPX4↑ Oa mice + D-mannose + Ad-Epas1 → HIF-2α↑, MDA↑, GPX4↓ OA mice + D-mannose + Ad-Epas1 + Fer-1 → cartilage destruction↓, MDA↓, HIF-2α↓, GPX4↑ [43]	Chondrocytes + IL-1β + D-mannose → MMP-3↓, MMP-13↓, PTSG2↓, HIF-2α↓ [43]
Ferrostatin (Fer)-1		Chondrocytes + IL-1β + Fer-1 → cell viability↑, collagen II↑, GPX4↑, ROS↓, MDA↓, TNF-α↓, SND1↓ [28]
Ferrostatin (Fer)-1		Fer-1 in mild OA → cell viability↑, GPX4↑, SLC7A11↑, MMP-13↓, ACSL4↓, p53↓ [44]
Forkhead box O 3 (FoxO3)		FoxO3 knocked down in mice chondrocytes → MMP-13↑, collagen II↓ Upregulation FoxO3 in IL-1β cells → ECM degradation↓, lipid peroxidation↓, ROS↓, iron concentration↓, SLC7A11↑, GPX4↑ Upregulation FoxO3 in cells treated with erastin → NF-kB↓, MAPK↓ [45]
G-protein coupled receptor 30 (GPR30)	Mice + DMM + G1 → OARSI↓ [46]	
Gamma-Oryzanol (β-Ory)		Rat chondrocytes + γ-Ory → Nrf2 movement into nucleus↑, presence HO-1 in cytoplasm↑, breakdown Nrf2↓ [47]
Heat shock protein family A member (HSPA5)		Mice chondrocytes + Ad-HSPA5 → GPX4↑, ROS↓, TNF-α↓, MDA↓, iron concentrations↓ Chondrocytes + sh-GPX4 → GPX4↓, ROS↑, TNF-α↑, MDA↑, iron concentrations↑ [28]
Icariin (ICA)	Mice + icariin → iron concentrations↓, bone loss↓ [48]	Chondrocytes + icariin → iron concentrations↓ [48]
Kukoamine A	OA mice + kukoamine A → loss of articular cartilage tissue↓, loss of cartilage matrix staining↓, MMP-1↓, MMP-3↓, COX2↓ [49]	Chondrocytes + IL-1β + kukoamine A → MDA↓, PGE2↓, MMP-1↓, MMP-3↓, iron concentration↓, translocation NF-kB p65 to nucleus↓, GSH↑, Nrf2↑, HO-1↑, SIRT1 [49]
Liproxstatin-1	OA mice + Liproxstatin-1 → joint swelling↓ [50]	
Mesenchymal stem cells-derived exosomes (MSC-Exos)	OA mice + MSC-exos → TNF-α↓, INF-γ↓, IL-6↓, IL-1β↓, LDH↓, cell viability↑, GSH↑, GPX4↑, GOT1/CC2↑ [51]	Chondrocytes + exos → TNF-α↓, INF-γ↓, IL-6↓, IL-1β↓, LDH↓, iron accumulation↓, cell viability↑, GSH↑, GPX4↑, GOT1/CC2↑ [51]
Metformin	OA mice + metformin → OARSI↓, MMP-13↓, p53↓, GPX4↑, SLC7A11↑ Erastin + metformin → OARSI↓, MMP-13↓, p53↓, GPX4↑, SLC7A11↑ [52]	
miR-1	OA mice + agomir-1 → OARSI↓, MMP-13↓, aggrecan↑, COL2↑, GPX4↑ [53]	
Moderate mechanical stress	OA exercise group → joint swelling↓, cartilage damage↓, MMP-13↓, p53↓, NF-kB p65 signaling pathway↓, SLC7A11↑, GPX4↑, Nrf2↑ [54]	
Peroxisome proliferator activated receptor γ (PPARγ)		Rat chondrocytes + RSL3 + pioglitazone (PPARγ agonist) → GPX4↑, pink1↑, parkin↑, PTGS2↓, MDA↓ [55]
Plumbagin (PLB)	OA mice + PLB → OARSI↓, MMP-13↓, GPX4↑ [56]	H_2_O_2_ signaling pathway can trigger MAPK signaling pathway. PLB hinders MAPK activation. [56]
Ruscogenin	OA mice + ruscogenin → MMP-1↓, MMP-3↓, cartilage damage↓ [35]	Chondrocytes + IL-1β + ruscogenin → PGE2↓, MMP-1↓, MMP-3↓, MDA↓, iron concentration↓, GSH↑, GPX4↑, Nrf2↑, SLC7A11↑, HO-1↑ [35]
Sarsasapogenin	Rats + DMM + sarsasapogenin → cartilage degradation↓, MMP-13↓, collagen II↑, GPX4↑, SLC7A11↑, YAP1↑ [57]	Rat chondrocytes + IL-1β + sarsasapogenin → MMP-3↓, MMP-13↓, COX2↓, aggregan↑, GPX4↑, SLC7A11↑, collagen II↑, YAP1↑ [57]
Spermidine		Mice chondrocytes + IL1- β + spermidine → lipid peroxidation↓, MDA↓, NCOA4↓, FTH↑, GPX4↑, SLC7A11↑ [23]
Stearoyl-CoA desaturase (SCD1)	SCD1 knocked out in OA mice → GPX4↓, p53↑, mitochondria shrinking↑ [58]	
Tanshinone IIA (Tan IIA)		Mouse chondrocytes + LPS + Tan IIA → ROS↓, MDA↓, iron concentration↓, GSH↑, GPX4↑ Chondrocytes + erastin + Tan IIA → cell viability↑, MMP-13↓ [34]
Theaflavin-3,3′	OA mice + erastin + theaflavin-3,3′ → cartilage damage repaired and reversed compared to OA + erastin [32]	
Transient receptor potential vanilloid 1 (TRPV1)	OA mice + TRPV1 agonist → chondrocytes↑, NCOA4↓ GPX4↓ in mice → loss of TRPV1′s anti-ferroptotic effect in OA cartilage [59]	Chondrocytes + oxidative stress reducer + TRPV1 agonist → cell viability↑, RSL3↓, ROS↓, lipid peroxidation↓, iron concentrations↓ [59]

**Table 2:** Ex vivo studies on ferroptosis in rheumatoid arthritis or osteoarthritis. Data derived from human cells confirm the findings from rodent FLSs and chondrocytes, as summarized in Table 1. It also displays ferroptosis-related biomarkers found in FLSs and chondrocytes of patients suffering from RA or OA. Disease-derived cells demonstrate augmented ferroptosis markers without further in vitro stimulation when compared to controls. ↑: upregulated; ↓: downregulated; →: results in.

Experiments in Humans	FLSs	Chondrocytes
**Detection of indicators for ferroptosis in arthritis**		
ACSF2, AURKA, EGFR, KLHL24 biomarkers ferroptosis in OA		30 samples of OA patients and 28 controls [60]
Increased ROS levels	RA-FLS, peripheral blood mononuclear cells from RA patients → ROS↑ in co-cultured RA-FLS and peripheral blood mononuclear cells [61]	ROS detection in mild and severe OA regions of OA patients [62]
Iron accumulation		Iron concentrations↑, transferrin expression↓, total iron binding capacity↓ in OA patients [39]
Lipid peroxidation		
LPCAT3 and PGD as possible diagnostic markers for OA		Human tibial plateau samples from 40 OA and 10 controls [63]
PTGS2, ENO1 and GRN as potential ferroptosis-related biomarkers	Synovial tissue from 15 RA patients and 7 controls [64]	
**Inducer of ferroptosis:**		
Acyl-CoA synthetase long-chain family member 4 (ACSL4)		ACSL4 silenced in chondrocytes →LDH↓, ROS↓, MDA↓, MMP-13↓, iron concentration↓, cell viability↑, GPXP4↑, GSH↑ [65]
CA-074Me	RA-FLS + CA-074Me → lipid oxidation rate↑, iron concentration↑, PTGS2↑, FTH1↓, SLC7A11↓, GPX4↓ [66]	
Dexamethasone		Healthy chondrocytes stimulated with dexamethasone → ROS↑, Akt↑, FoxO3↑ [33]
Erastin		Chondrocytes + erastin → cell viability↓, SLC7A11↓, FTH1↓, GPX4↓, cytotoxicity↑, TRPM7↑, ACSL4↑, COX2↑ [22]
Galectin-1 derived peptide (G1dP3)		MH7A cells + TNF-α and G1dP3 → ROS↑, iron concentration↑ p53↑, GSH/GSSG↓, GPX4↓, SLC7A11↓ [67]
Glycine	RA-FLS + glycine → S-adenosyl-methionine↑, methylation of GPX4 promotor↑, GPX4↓ [68]	
IL1-β		Chondrocytes + IL-1β → ATF3↑, TFRC↑, ROS↑, CXCL2↓, JUN↓ [69]
Lipopolysaccharide (LPS)	RA-FLS + LPS → ROS↑, ESCRT III↑, GPX4↓, SLC7A11↓ [70]	
MiR-181b		Chondrocytes + erastin → miR-181b↑ MiR-181b inhibited in chondrocytes → p53↓, MMP-13↓, TFR1↓, SLC7A11↑, GPX4↑, FTH1↑, collagen II↑ [71]
Nuclear receptor coactivator 4 (NCOA4)	NCOA4 knocked down + LPS → PTGS2↓, iron concentration↓, cell viability↑ LPS RA-FLS under hypoxia → HIF-1α↑, FTH1↑, PTGS2↓, NCOA4↓, ROS↓, iron concentration↓ [54]	Chondrocytes + IL-1β and NCOA4 knocked down → ACSL4↓, p53↓, iron concentration↓, ROS↓, MDA↓, GPX4↑, GSH↑, cell viability↑ [72]
OA-FLS exosomes	Exosomes derived from OA-FLS → miR-19b-3p↑ [27]	
Piezo1		OA chondrocytes + mechanical stress → piezo1↑, GPX4↓ [26]
Proinflammatory cytokines (IL-6, IL-1β, TNFα, IFN γ)	RA-FLS + TNF-α or IL-6 → absorption of transferrin-bound iron↑ RA-FLS + IL-1 or interferon γ → no significant effect [73]	
RAS-selective lethal 3 (RSL3)	RA-FLS + RSL3 → lipid peroxidation↑, iron concentration↑, FTH1↓, SLC7A11↓, SLC2A3↓, GPX4↓ [74]	
Specificity protein 1 (Sp1)		Chondrocytes + IL-1β → Sp1 Sp1 silenced in chondrocytes → Sp1↓, ACSL4↓ Sp1 overexpressed in chondrocytes → ACSL4↑ [65]
Sterol carrier protein 2 (SCP2)		Chondrocytes of OA patients → SCP2↑, MDA↑, iron concentration↑, lipid peroxidation↑, GPX4↓ Chondrocytes + SCP2 inhibitor → cell viability↑ [29]
Sulfasalazine	RA-FLS + sulfasalazine → p-PI3K/PI3K↓, p-AKT/AKT↓, p-ERK1/2/ERK1/2↓, p53↑ [30]	
Wasp venom (WV) I and WVII	MH7A cells + TNF-α and WVI or WVII → IL-1β↓, IL-6↓, JAK/STAT signaling pathway↓, ROS↑, GPX4↓ [75]	
Yin Yang 1 (YY1)	RA-FLS + LPS + SIRT1 + YY1 → ROS↑, iron concentration↑ [76]	
**Inhibitor of ferroptosis:**		
Baicalin		Chondrocytes + IL-1β + baicalin → cell viability↑, GPX4↑, SLC7A11↑, Nrf2↑, iron concentration↓, p53↓, ACSL4↓, ROS↓, MDA↓ [77]
Endosomal sorting complex required for transport (ESCRT)-III	Subunit of ESCRT-III knocked down → ROS↑, GPX4↓, SLC7A11↓ [70]	
Enolase 1 (ENO1)	ENO1 knocked down in RA-FLS → ROS↑, iron concentration↑, cell mortality↑, ACO1↑ [64]	
Epigallocatechin-3-gallate-based nanodrugs (ES NDs)		Chondrocytes + H_2_O_2_ + ES NDs → ROS↓, iron concentration↓, MDA↓, ACSL4↓, GPX4↑, FTH1↑ [62]
Ferrostatin (Fer)-1		Chondrocytes + IL-1β + Fer-1 → cell viability↑, cell proliferation↑, GPX4↑, SLC7A11↑, ROS↓, MDA↓, iron concentration↓, ACSL4↓, p53↓ [24]
G-protein coupled receptor 30 (GPR30)		Chondrocytes + erastin + G1 → cell viability↑, FTH1↑, GPX4↑, YAP1↑, ROS↓, lipid peroxidation↓ YAP1 knocked down → protective effects of G1↓ [46]
Icariin (ICA)	RA-FLS + LPS + ICA → cell death↓, iron concentration↓, GPX4↑, RA-FLS + RSL3 + ICA → Xc-/GPX4↑ [78]	
Long noncoding RNA (lncRNA) maternally expressed 3 (MEG3)		Chondrocytes + erastin + siMEG3 → SLC7A11↓, GPX4↓, cell viability↓, MDA↑, miR-885-5p↑ lncRNA MEG3 upregulated in chondrocytes → miR-885-5p↓, SLC7A11↑, GPX4↑ Chondrocytes + erastin + MEG3 → MDA↓, cell viability↑ OA samples → lncRNA MEG3↓, SLC7A11↓, miR-885-5p↑ [79]
miR-1		OA cartilage samples → miR-1↓, CX43↑ OA chondrocytes + miR-1 → cell proliferation↑, aggrecan↑, COL2↑, MMP-13↓, CX43↓ Chondrocytes + CX43 → aggrecan↓, COL2↓, MMP-13↑ [53]
Moderate mechanical stress		Chondrocytes + IL-1β + CTS → MMP-3↓, MMP-13↓, p53↓, NF-kB p65 signaling pathway↓, SLC7A11↑, GPX4↑, Nrf2↑ [80]
Puerarin		Chondrocytes + IL-1β + puerarin → cell viability↑, IL-1β↓, IL-6↓, TNF-α↓ [81]
Semaphorin 5A	RA-FLS + semaphorin 5A → PI3K/AKT/mTOR signaling pathway↑, GPX4↑ [82]	
Sirtuin 1 (SIRT1)	RA-FLS + LPS and SIRT1 was overexpressed → cell viability↑, ROS↓, iron concentration↓ [76]	
Theaflavin-3,3′		Chondrocytes + erastin + theaflavin-3,3 → ROS↓, iron concentration↓, FTH1↑, GPX4↑, SLC7A11↑, Nrf2↑, Keap1↑ [32]

## Abbreviations

**Abbreviation**	**Name**	**Description**
4-HNE	4-hydroxynonenal	Triggers inflammation
γ-Ory	Gamma-oryzanol	Substance found in rice bran oil
AA	Arachidonic acid	Type of omega-6 polyunsaturated fatty acid, primarily bound to ACSL4 through thioesterification process
ACLT	Anterior cruciate ligament transection	Used to induce OA in animal models.
ACO1	Aconitase 1	Cytosolic regulatory protein, monitors iron levels
ACPA	Anti citrullinated protein antibodies	Sustains inflammation in RA; connection between presence of ACPA and development of bone erosions and pain in RA
ACSF2	Acyl-CoA synthetase family member 2	Differentially expressed FRG
ACSL4	Acyl-CoA synthetase long-chain family member 4	Takes part in biosynthesis and remodeling of PE, facilitating PUFA activation, influencing transmembrane characteristics of PUFAs
AIF-M2	Apoptosis-inducing factor—mitochondria-associated	A protein that suppresses ferroptosis
Akt	Protein kinase B	A serin/threonine kinase
ALOX12	Arachidonate 12-lipoxygenase	Belongs to mammalian lipoxygenase family
ARE	Antioxidant response elements	Is activated by Nrf2
ATF3	Activating transcription factor 3	Is a key ferroptosis-related gen
AURKA	Serine/threonine-protein kinase 6	Is a ferroptosis-related biomarker in OA
AZ	Acetyl zingerone	Recently discovered antioxidant compound from curcumin
BCA	Biochanin A	Newly discovered bioactive compound from Huangqi (a plant)
BMPR2	Bone morphogenetic protein receptor type 2	An upregulated gene in PsA synovium
BzATP	2′(3′)-O-(4-Benzoylbenzoyl) adenosine 5-triphosphate	A ferroptosis agonist
CA-074Me	CA-074-methyl ester	Can cause ferroptosis, cathepsin B inhibitor
CAT	Capsiate	A metabolite produced by intestinal microorganisms
CCR2	C-C chemokine receptor type 2	A chemokine receptor
CDKN1A	Cyclin-dependent kinase inhibitor 1A	A ferroptosis-related gene
CIA	Collagen-induced arthritis	Collagen-induced arthritis in a mouse model
CISD1	CDGSH iron sulfur domain 1	A key ferroptosis regulator linked to PsA
CLEC2B	C-type lectin domain family 2 member B	A hub gene in PsA
COL2	Collagen type II	Decreased in ferroptotic cells.
COX2	Cyclooxygenase 2	Increased during ferroptosis as it is a ferroptosis regulator
CTSB	Cathepsin B	A potential biomarker for RA, associated with ferroptosis
CX43	Connexin 43	The target gene of miR-1
CXCL	Chemokine ligand	Chemokines; subfamily strongly linked with tumors and inflammatory conditions
DEGs	Differentially expressed genes	Expression enriched in mitochondria-related pathways
DMM	Destabilization of the medial meniscus	Animal models of OA established by DMM surgery
DMT1 = SLC11A2	Divalent metal transporter 1	Facilitates transfer of Fe^2+^ from endosome to labile iron pool within cytoplasm
Drp1	Dynamin-related protein	Protein associated with the process of mitochondrial division
EGFR	Epidermal growth factor receptor	Ferroptosis-related biomarker in OA
EGR1	Early growth response 1	A ferroptosis-related gene
ENO1	Enolase 1	A hub gene in ferroptosis
ERK	Extracellular signal-regulated kinase	Serin/threonine kinase
ES NS	Epigallocatechin-3-gallate-based nanodrugs	Reduce OA induced by ferroptosis
Fer	Ferrostatin	Ferroptosis inhibitor
FoxO	Forkhead box O	FoxO signal pathway important in ferroptosis
FPN = SLC11A3	Ferroportin	Protein for iron export
FRG	Ferroptosis-related genes	Used to categorize AS into two subtypes
FSP1	Ferroptosis-suppressor-protein 1	A protein that suppresses ferroptosis
FTH	Ferritin heavy chain	Is used to create ferritin
FTL	Ferritin light chain	Is used to create ferritin
G1dP3	Galectin-1-derived peptide	Anti-inflammatory and anti-proliferative properties in RA-FLS
GLX	GLX351322	A new selective NOX4 inhibitor
GOT1	Glutamic-oxaloacetic transaminase 1	Catalyzes reversible transfer of α-amino group between aspartate and glutamate
GSH	Glutathione	Plays a role in reducing ROS
GPR30	G-protein coupled receptor 30	Inhibits YAP1 and suppresses ferroptosis in chondrocytes
GPX4	Glutathione peroxidase 4	A pivotal enzyme in cellular defense against lipid peroxidation
GRN	Granulin	A ferroptosis-related gene
GSK-3β	Glycogen synthase kinase 3 beta	Inhibiting GSK-3β enhances ability to resist ferroptosis in CIA mice
GsMTx4	Grammostola mechanotoxin #4	A spider venom that inhibits piezo1
H1299 cells	Epithelial like cell	This cell line stably expresses p53
HLA	Human leukocyte antigen	Different types of HLA play important in different forms of arthritis
HO	Heme oxygenase	Antioxidant downstream protein of Nrf2, associated with ferroptosis
HSPA5	Heat shock protein family A member	Stabilizes GPX; identified as a ferroptosis inhibitor
HSPB1	Heat shock protein beta 1	A possible regulator for ferroptosis
ICA	Icariin	Active component found in Herba epimedii; possesses antioxidative properties and functions as an antiosteoporotic agent
IKE	Imidazole ketone erastin	A ferroptosis inducer by inhibiting the system Xc-
IL	Interleukin	Pro-inflammatory cytokines
IRE	Iron-responsive element	Upregulation of TfR1, downregulation of FTH and FPN with IRPs
IREB2	Iron-responsive element binding protein 2	By suppressing this protein, erastin-induced ferroptosis is restrained
IRPs	Iron metabolic regulating proteins	Upregulation of TfR1, downregulation of FTH and FPN with IRE
iTRAQ	Isobaric tags for relative and absolute quantification	An isobaric labeling technique employed in quantitative proteomics via tandem mass spectrometry for assessing the quantity of proteins originating from various sources within a singular experiment
JAK/STAT	Januskinase/signal transducers and activators of transcription	Inhibiting this signaling pathway, the IL-1β and IL-6 levels in TNF-α stimulated MH7A cells are reduced
JNK	c-JUN N terminal kinase	Inhibiting JNK-JUN-NCOA4 axis attenuated development of post-traumatic OA
JUN	Transcription factor JUN	A hub gene involved in ferroptosis
Keap1	Kelch-like ECH associated protein-1	A negative regulator of Nrf2
LDH	Lactate dehydrogenase	For classifying ferroptosis intensity
LOX	Lipoxygenases	Iron-containing enzymes that do not require heme, producing lipid messengers responsible for modulating cellular inflammation by inhibiting the oxidation of PUFAs
LPCAT3	Lysophosphatidylcholine acyltransferae 3	Takes part in biosynthesis and remodeling of PE, facilitating PUFA activation, influencing transmembrane characteristics of PUFAs
LPS	Lipopolysaccharide	A large glycolipid
m6A	N6-methyladenosine	A rare nucleoside of RNA
MAPK	Mitogen-activated protein kinase	Plays important role in ferroptosis through nuclear factor (NF)-kB/mitogen-activated protein kinase (MAPK) signaling pathway
MDA	Malonaldehyde	Is a derivate of PUFAs
MEG3	Maternally expressed 3	A long non-coding, imprinted RNA gene expressed from maternal allele
METTL3	Methyltransferase-like 3	A major methyltransferase that catalyzes formation of N6-methyladenosine (m6A) in mRNA
MH7A	Human rheumatoid arthritis synovial cell line	Used in research
MMA	Methyl methacrylate	A colorless liquid with an acrylic odor
MMP	Matrix metalloproteinase	Part of the metzincin family characterized by zinc-containing multidomain structures; function as proteases
mPEG-TK	Polyethylene glycol ketone mercaptan	Forms nanoparticles with GLX
MTX	Methotrexate	A medication usually used for treating RA
NCOA4	Nuclear receptor coactivator 4	A specific cargo receptor, facilitating autophagic ferritin breakdown
NF-kB	Nuclear factor—kappaB	Plays important role in ferroptosis through nuclear factor (NF)-kB/mitogen-activated protein kinase (MAPK) signaling pathway
NLRP3	NLR family pyrin domain containing 3	Is an inflammasome
NOX4	NADPH oxidase 4	A catalytic subunit of the NADPH oxidase complex
Nrf2	Nuclear factor erythroid 2-related factor 2	Controls ferroptosis-related gene expression
OARSI	Osteoarthritis Research Society International	Score is used to classify the severity of arthritis
P21	Cyclin-dependent kinase inhibitor 1A	Contributes to maintaining cell balance by preventing cell death through apoptosis and acts as a ferroptosis suppressor
P53	Tumor protein p53	Can induce ferroptosis via GPX4-dependent pathway or GPX4-independent pathway, tumor suppressor gene
P65	Nuclear factor NF-kappa-B p65 subunit	A transcription factor
PBS	Phosphate-buffered saline	Used in research as it is a buffer solution
PCBP	Poly-(rC)-binding protein	Act as chaperones
PE	Phosphatidylethanolamine	Functions as key phospholipid inducing ferroptosis in cells
PGD	Phosphogluconate dehydrogenase	A gene associated with ferroptosis
PGE2	Prostaglandin E2	It is increased during ferroptosis
Piezo1	Piezo-type mechanosensitive ion channel component 1	Converts diverse mechanical stimuli into electrochemical signals
PI3K	Phosphatidylinositol3-kinase	A transferase
Pink1	PTEN-induced kinase 1	Protein kinase, Pink1/Parkin-dependent mitophagy pathway has a protective effect on chondrocytes
PLB	Plumbagin	Has anti-inflammatory, antioxidant, and anti-cancer characteristics
PPARγ	Peroxisome proliferator activated receptor γ	Transcription factor activated by ligands; plays crucial role in controlling expression of multiple genes necessary for regulating lipid and glucose metabolism
PTGS2	Prostaglandin endoperoxide synthase 2	Biomarker associated with ferroptosis
PUFAs	Polyunsaturated fatty acids	Their characteristics play a vital role in preserving the fluidity of cell membranes, suppressing inflammatory mechanism, and reducing the release of proinflammatory cytokines by macrophages; a pivotal element for ferroptosis
PVA	Polyvinyl acetate	An aliphatic rubbery synthetic polymer
RA-FLS	Fibroblas-like synoviocytes in rheumatoid arthritis	Contribute to damage and inflammation within joints
RANKL	Receptor activator of nuclear factor kB ligand	A pivotal factor for the creation of osteoclasts
Ras	Rat sarcoma (virus)	Protein family involved in several pathways of cellular activity, often reregulated in cancer
ROS	Reactive oxygen species	Accumulation of ROS triggers ferroptosis
RSL3	RAS-selective lethal 3	A ferroptosis inducer
SCD1	Stearoyl-CoA desaturase	Induces ferroptosis in chondrocytes
SCP2	Sterol carrier protein 2	Non-specific lipid-transfer protein found in multiple tissues and cells, plays a significant role in different diseases
shRNA	Short hairpin RNA	Can be used to artificially silence genes using RNA interference
SIRT1	Sirtuin 1	Acts as a histone deacetylase, is highly conserved, relied on nicotinamide adenine dinucleotide (NAD+), holds pivotal functions in various cellular processes
SLC2A1	Solute carrier family 2 member 1	Transmembrane carrier for dehydroascorbic acid and glucose, through its activation the progression of ferroptosis is impeded
SLC2A3	Solute carrier family 2 member 3	Reducing the expression of SLC2A3 triggers ferroptosis in RA-FLS
SLC3A2	Solute carrier family 3 member 2	A subunit of system Xc-
SLC3A2L	4F2 cell-surface antigen heavy chain	LPS stimulation decreases the expression SLC3A2L
SLC7A11	Solute carrier family 7 member 11	A subunit of system Xc-
SND1	Staphylococcal nuclease domain containing 1	RNA-binding protein
SP1	Specificity protein 1	Belongs to the SP/KLF family of transcription factors, situated within the nucleus, participates in the regulation of numerous genes within mammalian cells, can interact with various proteins
STEAP3	STEAP3 Metalloreductase	Converts Fe^3+^ to Fe^2+^
System Xc-	Cystine/glutamate transporter	Amino acid antiporter, forms a crucial part of the cellular antioxidant system
TBHP	Tert-Butyl hydroperoxide	Can induce inflammation
TfR 1	Transferrin receptor 1	Upregulation of TfR1 leads to intracellular iron accumulation
TFRC	Transferrin receptor	A key ferroptosis-related gene
TGF-β	Transforming growth factor-beta	Could increase susceptibility of FLSs to ferroptosis
TGFBR1	Transforming growth factor beta receptor 1	Is overexpressed in the synovium of PsA patients
TNF-α	Tumor necrosis factor alpha	Inflammatory cytokine generated by macrophages in response to acute inflammation, triggers various signaling pathways within cells
TRPM7	Transient receptor potential melastatin 7	Blocking TRPM7 in chondrocytes shielded from ferroptosis
TRPV1	Transient receptor potential vanilloid 1	Its activation might safeguard chondrocytes from ferroptosis
VDAC	Voltage-dependent anion channel	Is involved in ferroptosis
VEGF	Vascular endothelial growth factor	Plays a crucial role in regulating the formation of blood vessels during vascular development and postnatal angiogenesis, is indispensable for the development and healing of bones
WGCNA	Weighted gene co-expression network analysis	A computational technique that evaluates the connections among measured gene transcripts, identifies groups of genes that are co-expressed in a clinically relevant manner and investigates pivotal genes within diseases pathways from the prospect of systems biology
WNT3A	Wnt family member 3A	Is overexpressed in the synovium of PsA patients
XO	Xanthine oxidase	Generates uric acid
YAP1	Yes-associated protein 1	An augmentation of this protein inhibits ferroptosis in OA
YY1	Yin Yang 1	Is a member of Gli-Kruppel zinc finger proteins, plays a role in inhibiting and activating gene promotors
